# The Added Value of Bloodpool SPECT/CT in Painful Non-Operated Foot and Ankle Undiagnosed With Standard Three-Phase Bone Scintigraphy

**DOI:** 10.3389/fmed.2021.634419

**Published:** 2021-03-05

**Authors:** Cécile Cuvilliers, Xavier Palard-Novello, Clémence Pontoizeau, Pierre Meneret, Anne Devillers, Florence Le Jeune, Antoine Girard

**Affiliations:** ^1^Nuclear Medicine Department, Centre Eugène Marquis, Rennes, France; ^2^University of Rennes 1, Rennes, France

**Keywords:** bloodpool SPECT, bone scintigraphy, ankle, foot, early SPECT/CT

## Abstract

**Purpose:** To evaluate the interest of adding a bloodpool SPECT/CT to standard three-phase bone scintigraphy (BS) for etiological diagnosis of subacute and chronic lower extremity pains.

**Methods:** We prospectively included patients addressed for pain of lower extremities lasting for at least 6 weeks, without previous surgery. They underwent a standard three-phase BS including late phase SPECT/CT, modified with an additional bloodpool SPECT/CT acquisition. Two independent physicians interpreted the images provided by both protocols. Diagnostic conclusion, diagnostic confidence, and interrater agreements were compared.

**Results:** One hundred and eighteen lower extremities from 113 patients were analyzed (71 men, median age of 53 years). Adding bloodpool SPECT/CT to standard three-phase BS changed diagnostic conclusions in 24.6% (29/118) of lower extremities. The modified protocol revealed at least one diagnostic conclusion explaining the pain in 89% of extremities, rather than 83.1% with the standard protocol (*p* = 0.02). Tendinopathies were diagnosed in 12.7% of lower extremities, rather than 4.2% with standard BS (*p* = 0.002). Adding bloodpool SPECT/CT substantially increased overall confidence of each reader (*p* < 0.001). Inter-reader agreement was not significantly impacted.

**Conclusion:** Adding bloodpool SPECT/CT to standard three-phase BS impacted diagnostic conclusion in a quarter of the patients with painful lower extremities, notably by revealing significantly more tendonitis.

## Introduction

Subacute and chronic pains of foot and ankle are common reasons for healthcare encounter (1). Many causes can be responsible, such as osseous and articular pathology, as well as tendonitis, ligamentopathy, and complex regional pain syndrome (CRPS) with different therapeutic management. Identifying the underlying etiology of the pain can be challenging, even after detailed clinical examination and initial radiography. This frequently makes further imaging explorations necessary (2). Three-phase bone scintigraphy (3pBS) is an imaging method of choice to establish etiological diagnosis of painful lower extremities (3). The rise of late-phase single photon emission computed tomography (SPECT) has dramatically increased diagnostic performances of bone scintigraphy by enhancing its sensitivity (4), and its localization ability (5). Moreover, with the advent of hybrid imaging, specificity was increased by adding CT to SPECT. These technical developments provided substantial improvement in diagnosis and management of patients with pain, particularly regarding painful lower extremities (6).

Despite the major contribution of bone scintigraphy to diagnose osseous and articular pathologies, the cause of the pain remains unrevealed in many patients (3). To date, only a few studies investigated the role of bone scintigraphy to detect extra-osseous abnormalities, notably concerning foot and ankle pain (7, 8). Based on the standard bloodpool planar images, bone scintigraphy can reveal inflammatory process in extra-osseous tissues (9), while late-phase images appear normal. Verschueren et al. recently reported that bloodpool SPECT outperforms planar imaging in the assessment of painful total knee arthroplasty and improves information on prosthesis outcome (10). Considering the prevalence of extra-osseous pathologies (11) and the particularly complex anatomy of the feet and ankles (11, 12), we assumed that bloodpool SPECT/CT could be of interest when exploring subacute and chronic lower extremities pain and may detect extraosseous pathology unrevealed with standard 3pBS.

The aim of this study was to evaluate the interest of adding a bloodpool SPECT/CT to standard 3pBS for etiological diagnosis of subacute and chronic lower extremities pain, regarding diagnostic conclusion, inter-reader agreement, and diagnostic confidence.

## Materials and Methods

### Participants

We prospectively included from March 2019 to March 2020 consecutive patients referred to our center to perform a bone scintigraphy who matched the following inclusion criteria: minimum age of 18 years old, subacute (evolving for 6–12 weeks) or chronic (evolving for more than 3 months) foot and/or ankle pain, no previous surgery of the affected lower extremity, affiliation to national healthcare insurance. Exclusion criteria were local anti-inflammatory infiltration within the last month and intake of anti-inflammatory systemic drug within the past week. This study performed in routine care was reviewed and approved by the University Hospital of Rennes ethics committee (approval n°19.99 −2). According to the current regulations of the European Union for prospective observational studies, participants received written information and did not object to participate. Clinical information was obtained from both the examination request letter written by the corresponding practitioner and a standardized questionnaire filled with each patient.

### Imaging Procedures

All patients received bone scintigraphy according to the guidelines of the European Association of Nuclear Medicine, consisting of planar 2 min early dynamic and bloodpool acquisitions centered on feet/ankles, and late-phase planar whole-body and SPECT/CT images of the lower extremities. The radiopharmaceutical used was hydroxy-methylene-diphosphonate (HMDP) labeled with 99m-technetium. Bloodpool planar images were acquired systematically in anterior-posterior 5 min projection, and additional projection on request from the nuclear medicine physician. In addition, a bloodpool SPECT/CT centered on feet and ankles was performed 7–14 min after injection. Images were acquired on hybrid SPECT/CT system (Discovery 670, GE Healthcare, or Symbia T16, Siemens Healthineers). Both bloodpool and late-phase SPECT/CT acquisitions were performed with the step-and-shoot acquisition system on the Discovery camera (30 projections of 20 s, yielding a total acquisition time of 10 min) or the continuous acquisition system on the Symbia camera (30 projections of 15 s, total acquisition time of 7 min and 30 s). SPECT data were reconstructed using the Recon Flash 3D on Discovery camera, and an ordered subset expectation maximization on the Symbia camera (128 × 128 matrix, pixel size of 4.4 mm in every axis). A low-dose CT was performed during bloodpool phase (Discovery: voltage 80 kV, 60 mAs, slice thickness and spacing 3,75 mm, iterative reconstruction, Symbia: 80 kV, 35 mAs, Care Dose collimation, slice thickness and spacing 5 mm, with reconstruction of 3 mm) and a diagnostic CT associated with late-phase SPECT (Discovery: 120 kV, maximum of 140 mAs with modulation, slice thickness 0.625 mm and spacing 0.5 mm, iterative reconstruction; Symbia: 130 kV, 120 mAs, slice thickness and spacing of 0.75 mm each 0.5 mm).

### Images Interpretation

Two nuclear medicine physicians, aware of clinical information, read independently all the images in two steps. A first interpretation was based on images provided by standard 3pBS (including angiographic phase, bloodpool planar imaging, and late-phase SPECT/CT), then, at least 3 months apart and blinded to prior interpretation, a second reading relied on images obtained with the modified protocol (i.e., with the addition of bloodpool SPECT/CT). In both steps, CT images from hybrid modalities were taken into account in the interpretation. For each interpretation step, each reader provided a description of abnormalities, and a diagnostic conclusion addressing the diagnostic issue with an overall confidence score on a 3-points scale (1: low confidence, 3: high confidence). For each reader, diagnostic conclusions were synthesized as CRPS in hot phase, CRPS in cold phase, fracture (or pseudoarthrosis), arthropathy (from distal tibiofibular joint to toes joints, including accessory sesamoid bones conflicts and osteochondral lesions of the talar dome), ligamentopathy (including ankle and tarsal ligaments and distal tibiofibular syndesmosis), tendinopathy, calcaneal spur, other diagnostic orientation, or no diagnostic orientation. Finally, on each step of interpretation, in case of discrepancy regarding the diagnostic conclusion, a common reading was made until a consensus was reached.

### Study Endpoints

The primary endpoint was the diagnostic conclusion provided by bone scintigraphy. The hypothesis of the study was that the use of bloodpool SPECT/CT provides a substantial change in diagnostic conclusion compared to standard 3pBS protocol (reveals more, less, or some other diagnoses). The secondary endpoints were the inter-rater agreement and the overall confidence. The hypotheses were that adding bloodpool SPECT/CT to standard protocol increases inter-rater agreement and overall confidence regarding for the diagnostic conclusions.

### Statistical Analysis

Statistical analysis was performed with the MedCalc^®^ version 12.5.0.0 (Medcalc Software, Ostend, Belgium). Distribution of continuous variables were presented as median [Interquartile range (IQR)]. Proportions for each kind of diagnostic conclusion provided by the consensus of experts were compared between standard and modified protocols using the McNemar's *t*-test. Scores for diagnostic confidence of both readers were compared between standard and modified protocols using a Wilcoxon signed test. Interrater agreements were assessed for each kind of diagnostic orientation using Cohen's kappa coefficient with 95% confidence intervals (95%CI) (values ≤0 as indicating no agreement, 0.01–0.20 as none to slight, 0.21–0.40 as fair, 0.41–0.60 as moderate, 0.61–0.80 as substantial, and 0.81–1.00 as almost perfect agreement). All statistical tests were two-tailed and statistical significance level was set at *p* < 0.05.

## Results

### Participants

**Figure 1:**
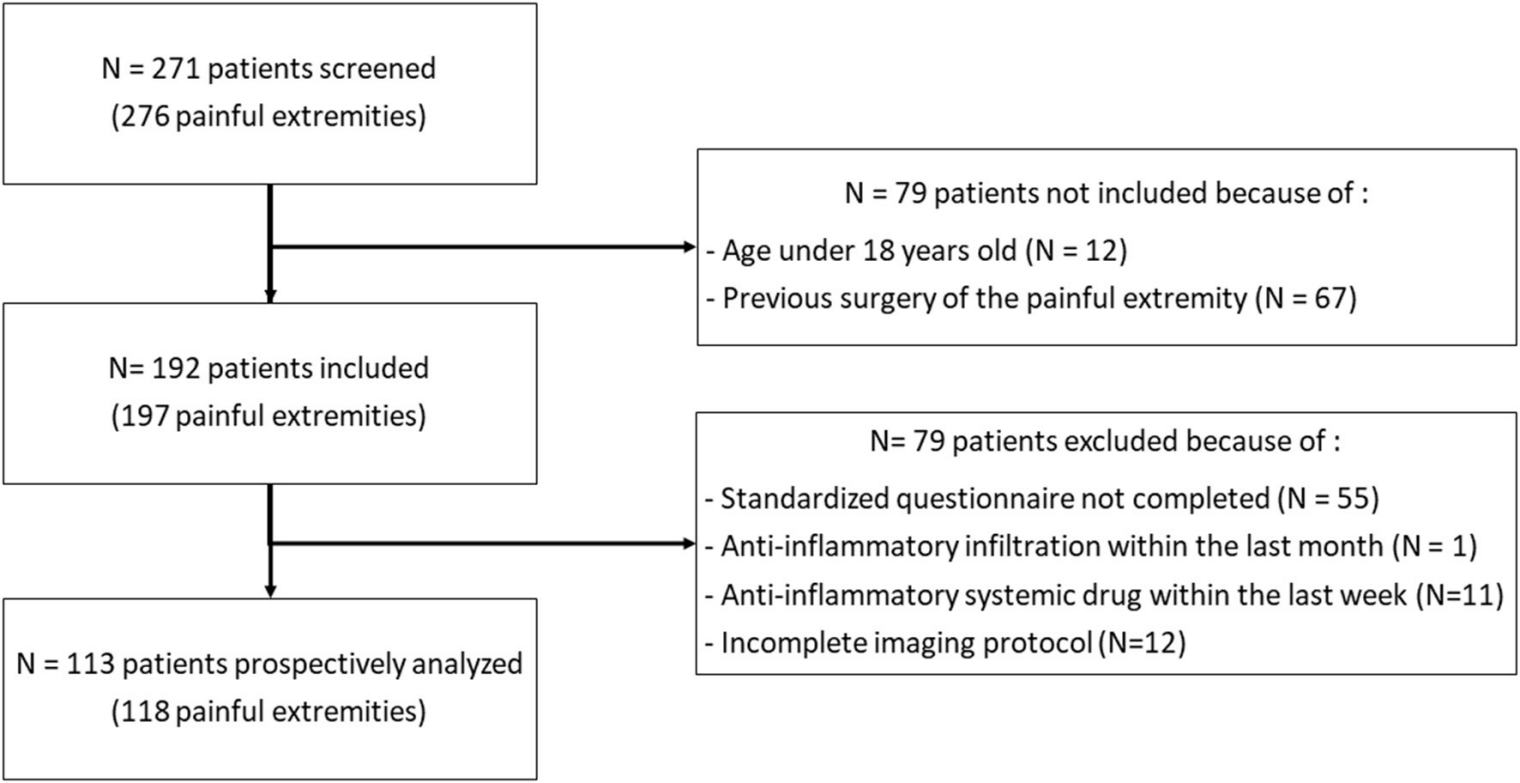
Flowchart of the study.

Two hundred and seventy-one patients were prospectively screened, 79 did not match inclusion criteria, and 79 were excluded. One hundred and eighteen lower extremities from 113 patients [71 men, median age of 53 years (IQR: 37–66)] were finally analyzed. The flowchart of participants is presented in Figure 1. The lower extremity pains lasted for a median duration of 14 weeks (7–40) with an intensity of 5 (4–7) on a 10-points visual analog scale. Thirty-six (31.9%) patients were suspected with CRPS by the corresponding clinician, and 59 (50%) reported a trauma. Characteristics of patients are presented in Table 1.

**Table 1:** Participant characteristics.

**Characteristic**	***N* (Proportion)**	**Median** **[Range]**
Participants	113	
Age *(years)*		53 [37, 66]
**Gender**		
Male	71 (63%)	
Female	42 (37%)	
**Pain**		
Left only	63 (55.8%)	
Right only	45 (39.8%)	
Bilateral	5 (4.4%)	
Duration *(weeks)*		14 [7, 40]
Intensity *(scale from 0 to 10)*		5 [4, 7]
Mechanical type only	28 (24.7%)	
Inflammatory type only	10 (8.9%)	
Mixed type	75 (66.4%)	
Suspicion of complex regional pain syndrome	36 (31.9%)	
Reported trauma	59 (50.0%)	
**Referred by**		
General practitioner	63 (55.8%)	
Specialist	50 (44.2%)	

### Diagnostic Conclusions

**Figure 2:**
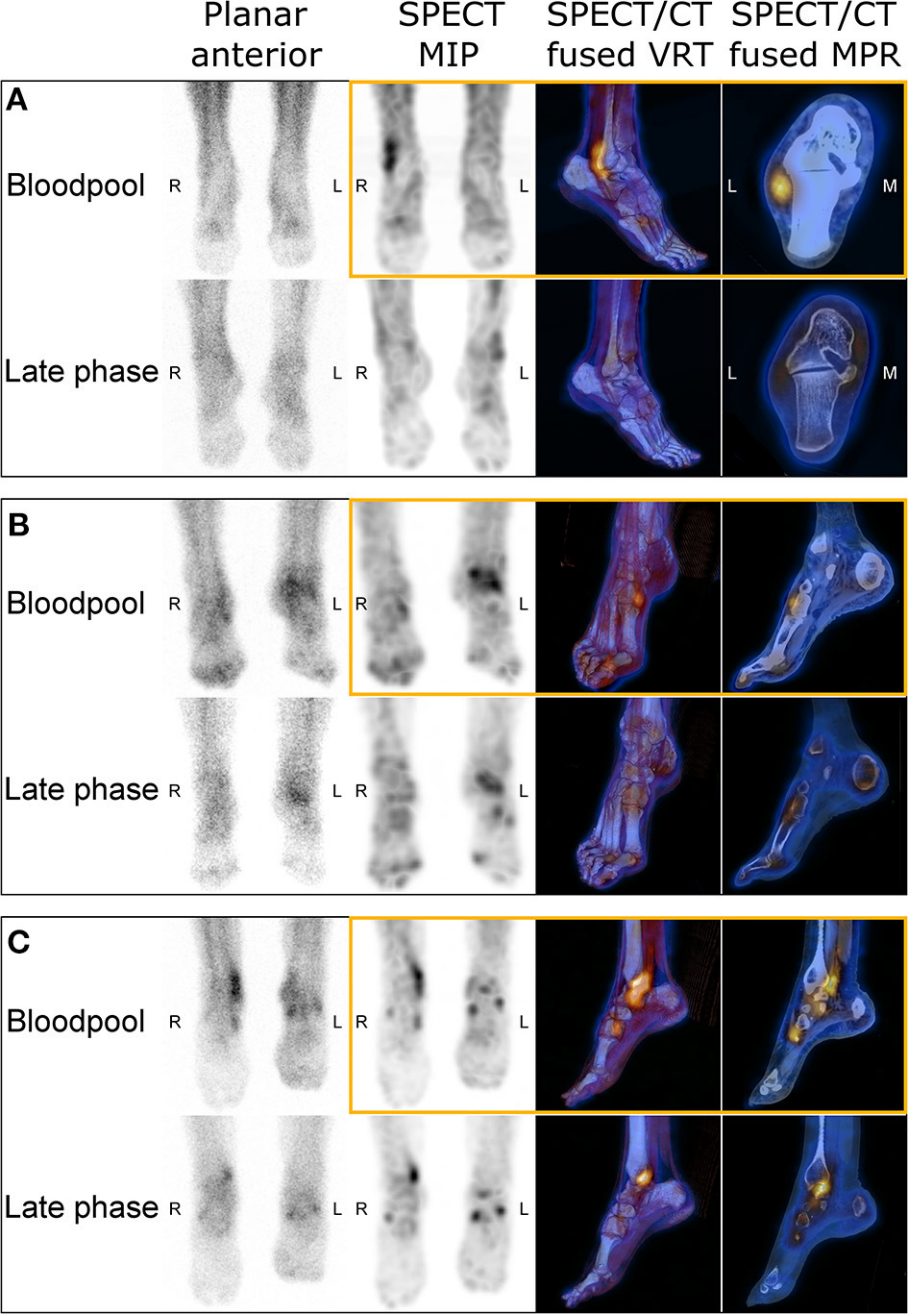
Images of three-phase bone scintigraphy from three patients with right lower extremity pain, including images provided by bloodpool SPECT/CT (in orange boxes). Patient **(A)** was a 62-year-old man referred for suspicion of right lateral malleolus stress fracture. Bloodpool SPECT/CT revealed intense uptake along peroneal tendons, highly suggesting the diagnosis of right peroneal tendonitis. Patient **(B)** was an 81-year-old woman with pain of the right ankle and anterior tarsus, suspected with stress fracture. Bloodpool SPECT/CT revealed focal uptake at the insertion of the anterior tibial tendon, leading to set the diagnosis of right tibial anterior tendonitis. Patient **(C)** was a 60-year-old woman, suffering from right medial ankle pain. Planar bloodpool and late phase SPECT/CT images were compatible with a posterior tibial tendonitis with a low diagnostic confidence. Bloodpool SPECT/CT substantially increased the diagnostic confidence for posterior tibial tendonitis and revealed intense focal uptake at the insertion of the anterior tibial tendon, evocative of associated right tibial anterior tendonitis.

Adding bloodpool SPECT/CT substantially impacted diagnostic conclusions in 29 out of 118 (24.6%) lower extremities. Diagnostic conclusions provided by both standard and modified imaging protocols are presented in Table 2. Whereas, 98 (83.1%) lower extremities were found with at least one diagnostic conclusion explaining the symptoms with standard 3pBS including late-phase SPECT/CT, 105 (89.0%) (*p* = 0.02) showed at least one diagnostic conclusion with the modified imaging protocol including supplementary bloodpool SPECT/CT. The only diagnostic category that detection was significantly impacted by adding bloodpool SPECT/CT was “tendinopathy,” revealed in 15 (12.7%) lower extremities with modified protocol, rather than five (4.2%) with standard 3pBS with late-phase SPECT/CT (*p* = 0.002). The bloodpool SPECT/CT revealed 12 additional tendons with pathological uptake (including two lower extremities with another tendonitis already visualized with the standard protocol): five posterior tibial tendons, three anterior tibial tendons, three calcaneal tendons, and one fibular tendon (Figure 2).

**Table 2:** Diagnostic conclusions and inter-reader agreement compared between the standard three-phase bone scintigraphy and modified protocol.

	**Diagnostic consensus** **Standard protocol** ***n (%)***	**Diagnostic consensus** **Modified protocol** ***n (%)***	***p*-value**	**Inter-reader agreement** **standard protocol** **Kappa *[95%C ^b^]***	**Inter-reader agreement** **modified protocol** **Kappa *[95%C ^b^]***
CRPS^a^ hot phase	8 (6.8%)	5 (4.2%)	0.25	0.64 [0.39, 0.88]	0.59 [0.27, 0.92]
CRPS^a^ cold phase	2 (1.7%)	3 (2.5%)	1.00	−0.01 [−0.03, 0.01]	0.49 [−0.11, 1.00]
Fracture	41 (34.7%)	42 (35.6%)	1.00	0.78 [0.66, 0.90]	0.79 [0.68, 0.91]
Calcaneal spur	3 (2.5%)	4 (3.4%)	1.00	0.66 [0.21, 1.00]	0.43 [0.028, 0.83]
Arthropathy	52 (44.1%)	57 (48.3%)	0.06	0.74 [0.61, 0.86]	0.58 [0.43, 0.72]
Ligamentopathy	17 (14.4%)	18 (15.3%)	1.00	0.66 [0.48, 0.85]	0.74 [0.57, 0.90]
Tendinopathy	5 (4.2%)	15 (12.7%)	0.002^*^	0.27 [−0.07, 0.61]	0.57 [0.32, 0.82]
Other	1 (0.8%)	1 (0.8%)	–	1.00 [1.00, 1.00]	1.00 [1.00, 1.00]
At least one diagnostic	98 (83.1%)	105 (89.0%)	0.02^*^	0.67 [0.49, 0.84]	0.63 [0.40, 0.85]

a CRPS, complex regional pain syndrome;

* , p value < 0.05;

b *CI, confidence interval*.

### Diagnostic Confidence

Overall confidence of each reader was significantly higher based on the modified protocol including bloodpool SPECT/CT than using the standard 3pBS with late-phase SPECT/CT only. The mean ± standard deviation overall diagnostic confidence was 2.0 ± 0.8 with the standard protocol, and 2.5 ± 0.7 with the addition of bloodpool SPECT/CT (*p* < 0.0001) for reader 1, and 1.9 ± 0.7 and 2.3 ± 0.8 (*p* < 0.0001), respectively, for reader 2.

### Inter-reader Reproducibility

Inter-reader agreement on diagnostic conclusion are presented in Table 2. The addition of bloodpool SPECT/CT to standard 3pBS did not significantly modify inter-reader reproducibility for any diagnostic conclusion category regarding the Cohen's kappa coefficient.

### Bloodpool Imaging Description

**Figure 3:**
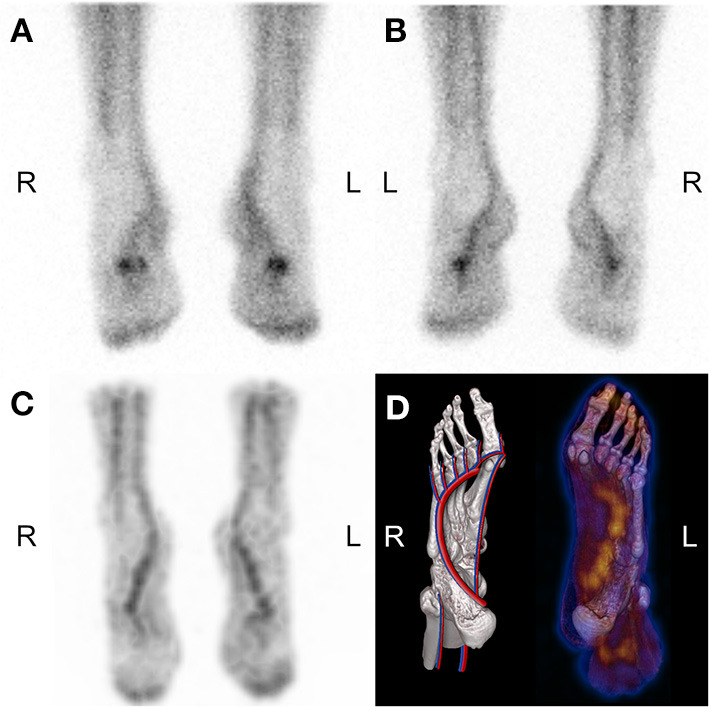
50-year-old man suffering from pain of third to fifth left metatarsus. Anterior **(A)** and posterior **(B)** planar bloodpool images suggested increased uptake in the projection of left and right midfoot. Bloodpool SPECT (**C**: MIP, **D**: SPECT fused VRT and anatomical drawing) refuted the “abnormality,” revealing physiological linear uptake on deep plantar arch.

To better understand how the addition of bloodpool SPECT/CT impacted the interpretation, here are reported the abnormalities described by the readers based on bloodpool images from the standard and the modified protocol, respectively. A total of 165 abnormalities from 93 lower extremities were described either on planar bloodpool and/or bloodpool SPECT images (Table 3). For 25 lower extremities, no bloodpool abnormality was found, neither on planar nor on SPECT images. One hundred and twelve (67.9%) bloodpool abnormalities from 61 (65.6%) feet or ankles were found on both planar and SPECT images, including 33 out of 112 (29.5%) abnormalities from 31 out of 61 (50.8%) extremities that were differently localized thanks to bloodpool SPECT/CT. The bloodpool SPECT revealed 44 (26.7%) uptake abnormalities from 33 (35.5%) extremities undetected with bloodpool planar images. Inversely, nine (5.4%) abnormalities from nine lower extremities (9.7%) were only reported based on planar images, but unseen with SPECT/CT. Five out of these nine abnormalities (55.6%) were unwell circumscribed increased uptake projecting on median midfoot on planar images. On bloodpool SPECT/CT images, these abnormalities matched linear uptakes following the lateral and deep plantar vessels, without any morphological abnormality (Figure 3).

**Table 3:** Bloodpool imaging description.

	**Abnormalities *n (%)***
Total	165 (100%)
Detectable with both planar and SPECT^a^ imaging	112 (67.9%)
*Identically localized*	*79 (70.5%)*
*Differently localized*	*33 (29.5%)*
Detectable with SPECT^a^ only	44 (26.7%)
Detectable with planar imaging only	9 (5.4%)
*Related to physiological activity in plantar vessels*	*5 (55.6%)*

a *SPECT, single photon emission computed tomography*.

## Discussion

In the last decade, the development of hybrid SPECT/CT substantially improved diagnostic performances of bone scintigraphy (13), particularly when imaging the extremities (14). Authors reported that late-phase SPECT/CT has high impact on therapeutic decisions for foot and ankle pain (15), notably if multiple pathologies are coexisting (16). However, to date, the role of bloodpool SPECT/CT has been poorly investigated, especially to explore lower extremities. Recently, authors related that bloodpool SPECT outperforms planar imaging in the assessment of painful total knee arthroplasty, by enhancing the inter-rater agreement and the overall confidence to localize inflammatory regions, and by providing relevant information on prosthesis outcome (10). The value of bloodpool SPECT or SPECT/CT has also been suggested when exploring non-oncological bone pain, improving diagnostic yield compared to planar imaging, showing additional lesions (17), and improving localization of abnormalities (18). Lastly, its benefit has been suggested for viability assessment in mandibular reconstruction (19), painful hip or knee prosthesis (20), and in rheumatoid arthritis (21).

The results of the present study clearly support the role of bloodpool SPECT/CT as a part of 3pBS in the etiological exploration of lower extremity pain. The addition of bloodpool SPECT/CT substantially changed the diagnostic conclusion in 29 out of 118 (24.6%) lower extremities. Using the modified protocol provided significantly less inconclusive exams, revealing at least one diagnostic conclusion explaining the symptoms in 105 out of 118 (89.0%) of painful lower extremities, rather than 98 out of 118 (83.1%) with standard 3pBS. This increased diagnostic yield was mainly explained by a better visualization of tendonitis, which was the only diagnostic category significantly impacted by adding bloodpool SPECT/CT. Modified protocol revealed tendonitis explaining the pain in 15 out of 118 (12.7%) lower extremities, rather 5 out 118 (4.2%) with standard protocol. Besides, the results show that adding bloodpool SPECT significantly improved overall diagnostic confidence of each reader.

Two properties of SPECT/CT compared to planar images can explain the best detection of tendonitis. First, the highest signal-to-noise ratio provided by SPECT may have enable the visualization of tendinous uptake unrevealed by standard bloodpool planar images (22, 23). Moreover, combining SPECT and CT is known to improve localization of scintigraphic abnormalities (16). Hybrid imaging can have led to the diagnosis of tendonitis in patients with slight and/or poorly localized uptake visualized on standard bloodpool planar images, that may initially have been considered non-specific. These observations are in line with those of Verschueren et al. showing a 20% improvement of rater confidence when using bloodpool SPECT compared to planar images, for localizing uptake on bloodpool images in painful total knee arthroplasty (10).

Painful lower extremity is a widespread cause of medical consultation and imaging procedure (1, 22). Extra-osseous pathologies, such as tendon and ligament lesions, are common causes of symptoms (12), for instance ligamentous ankle sprains involves 27,000 patients per day in the US (7, 22). Bone scintigraphy is an imaging procedure performed by almost all nuclear medicine centers across the world, mostly non-expert centers. According to our results, adding bloodpool SPECT/CT to standard 3pBS improves diagnostic yield for tendonitis and enhances overall diagnostic confidence. Thus, expanding this practice could lead to substantially decrease the number of patients with pain remaining unexplained and prevent unnecessary investigation. The time-consuming aspect of adding bloodpool SPECT/CT to standard 3pBS could hamper its use. In the present study, bloodpool SPECT/CT revealed at least as much uptake abnormalities than bloodpool planar images in 94.5% lower extremities, showing more uptake abnormalities in 35.5%. Five out nine (56%) uptakes seen as “abnormal” only on planar images were considered as linked with physiological vascular activity in the plantar vessels on bloodpool SPECT, ruling out some potential pitfalls. These results suggest that bloodpool planar acquisitions might be replaced with SPECT/CT without losing diagnostic value. By doing so, the extra time needed would not exceed 2.5–5 min per patient if only one planar projection was initially planned. When compared to the time spent for several planar projections, performing bloodpool SPECT can even be faster. Moreover, using bloodpool SPECT instead of planar images would prevent the need to perform several planar acquisitions requiring complex foot positioning (24) and thus improve the comfort for patient with painful lower extremities and limit uninterpretable images due to patients' movements. These results are in line with those of Verschueren et al. who recently concluded to a benefit of using bloodpool SPECT instead of planar images (10). Interestingly, in the present study there was no statistically significant difference between standard and modified protocol regarding inter-reader agreement about the diagnostic conclusions. In contrast, Verschueren et al. reported an increased inter-rater agreement for localizing tracer uptake with bloodpool SPECT (10).

Our study has some limitations that need to be considered. First, we did not have a follow-up for any of the patients evaluated since they were addressed by clinicians from other institutions, thus we did not get for any confirmation of diagnostic conclusion provided by the bone scintigraphy. This bias is found in most other studies working on soft tissue pathology, considering the lack of gold standard investigation (12). Nevertheless, all the diagnostic conclusions reported were consistent with the localization and the type of the pain. Secondly, only anterior and posterior projections were systematically acquired regarding planar bloodpool imaging, and acquisition of supplemental projections was left to the discretion of the physician. It could be discussed whether pseudo planar images may have replaced standard planar images. Nevertheless, we considered bone activity to be very low on bloodpool SPECT beginning 7 min after injection, and that it would not impact diagnostic conclusion. Finally, this study was designed to evaluate the interest of bloodpool SPECT/CT in foot and ankle pain. Included patients were selected to constitute a homogeneous sample, and we chose to not include patients with history of previous surgery of the painful lower extremity. We emphasize that bloodpool SPECT/CT might also be particularly useful in this population, that could be investigated in a forthcoming study. As an aside, technical considerations can be discussed since we performed this study using devices with NaI(Tl) scintillation detectors. The use of new devices with CZT semiconductor detectors could improve spatial resolution and sensitivity for both planar and SPECT images (25).

## Conclusions

The addition of bloodpool SPECT/CT to standard 3pBS for the exploration of painful lower extremities increased the diagnostic yield and enhanced the diagnostic confidence. No significant impact was observed regarding the inter-reader agreement. To improve its feasibility and the comfort of patients, our results suggest that bloodpool SPECT/CT could be performed instead of bloodpool planar images without missing meaningful information. This last point could be validated in a forthcoming study specially designed for.

## Data Availability Statement

The original contributions presented in the study are included in the article/supplementary material, further inquiries can be directed to the corresponding author/s.

## Ethics Statement

The studies involving human participants were reviewed and approved by University Hospital of Rennes ethics committee (approval n°19.99 −2). Written informed consent for participation was not required for this study in accordance with the national legislation and the institutional requirements.

## Author Contributions

AG and CC: study conception and design, screening and inclusion of patients, and bone scintigraphy analysis. CC: data collection. AG: statistical analysis. CC, AG, XP-N, PM, CP, AD, and FL: manuscript editing and reviewing. All authors contributed to the article and approved the submitted version.

## Conflict of Interest

The authors declare that the research was conducted in the absence of any commercial or financial relationships that could be construed as a potential conflict of interest.
